# A Stylometric Analog of the Fermi-Pasta-Ulam-Tsingou Problem: Combination of Human Bias and Long-Range Correlation Creates a Sort of Soliton

**DOI:** 10.3390/e28050574

**Published:** 2026-05-21

**Authors:** Kazuya Hayata

**Affiliations:** Sapporo Gakuin University, Ebetsu 069-8555, Japan; hayata@sgu.ac.jp

**Keywords:** FPUT problem, long-range correlation, Rényi entropy, Shannon entropy, modern translation, machine translation, *The Tale of Genji*

## Abstract

Revealing correlations for styles in texts has been an interesting topic, providing an example of trans-disciplinarity between physics and linguistics. Typical cases can be seen for sound correlations in verses as well as for word correlations in prose. Of these, long-range correlations are of particular interest because of their connection to the Markovian nature in human cognition. For a famous novel written in an archaic style of Japanese, we conduct an analysis of a series of *kanji* compounds in the text. Here, *kanji* is a Japanese name for Chinese characters. Specifically, we focus on the number (equivalent to the length) of a compound. Subsequently, the sequence of numbers is expanded into 6-bit binary codes (equivalent to 64 modes). Replacing each compound in the text with an oscillator in a string, for the chain of the *kanji* compounds, one can find an analogy with the so-called Fermi-Pasta-Ulam-Tsingou (FPUT) model. Comparative analyses for 16 modern translations by humans and machines show, without exception, a strong dominance for a particular mode and its stability bearing a remote resemblance to a soliton, and at the same time, reproduce a statistical property far from a sort of ergodicity. Furthermore, comparison between the human and machine translations shows that the entropy of the latter is higher than that of the former because machines are subjected to neither a psychological bias nor an inspection by editors. Lastly, in addition to the above translated texts, 6 codices of the classic are also analyzed, and their results are compared with those of the modern translations. Note that the original of the classic has not been found yet.

## 1. Introduction

**Figure 1:**
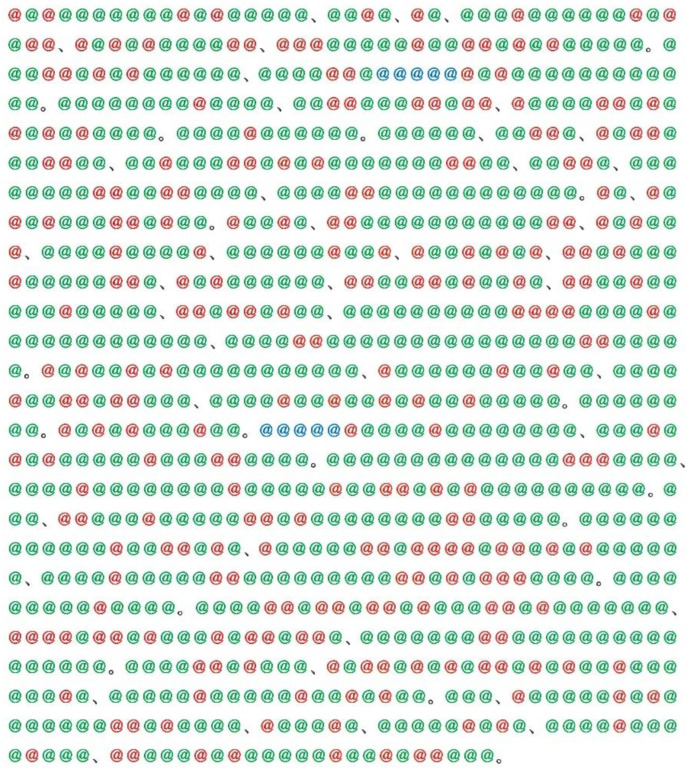
A bird’s-eye view of a typical Japanese writing, where red, green, and blue marks represent *kanji* (Chinese characters), *hiragana*, and *katakana*, respectively.

Quantitative studies have shown that, along with short-range correlations such as the nearest-neighbor and second-neighbor correlations, for natural languages, so-called long-range correlations might be ubiquitous in the sense that evidence of the phenomena has been found not only in verses but in general writings [1,2,3,4,5,6,7]. In contrast to alliteration and rhyming, correlations in the latter texts occur spontaneously, which can range from the opening to the last paragraph. That is, irrespective of a language being used, how to choose a word in the latter paragraph tends to depend more or less on which word was chosen in the former part of the entire text. In contrast to most languages whose texts are written in a single form of characters, for exceptional languages using multiple forms of characters, choices of their form are arbitrary, and consequently, writers are charged with their selection. For instance, writers of Japanese texts, a sample of which is given in Figure 1, should choose a form from the three kinds of characters consisting of *kanji*, *hiragana*, and *katakana*. Here, *kanji* is a Japanese name for Chinese characters, while the latter two are syllabics in a system of syllabary consisting of about 100 characters [8]. For instance, *toki* ‘when’, one of the frequent words in Japanese texts, has two choices of writing. One is the form written with a single *kanji* character, and the other is the one with two *hiragana* characters. It should be stressed here that the initial choice exerts a far-reaching influence on the final choice. In other words, if a writer (assuming male) chose a specific form, he must use the same form throughout the entire text. Furthermore, this rule should be applied to the entire volume of a novel. Even if he failed to preserve strict coherence in the use of characters, editors of his article will amend the form. It can be expected that such an apparently peculiar rule in the writing of Japanese texts results in preserving a far-reaching correlation in the style. For this reason, it is worth emphasizing that Japanese texts will provide a fascinating system for finding unknown properties of modern quantitative linguistics.

In this paper, we analyze a series of *kanji* compounds in a famous novel written in the classical style of Japanese. Specifically, in a number of modern translations for the first volume of *Genji Monogatari, ‘The Tale of Genji*’, we focus on the sequence of the number (*ν*) of characters in a *kanji* compound (*ν* = 1, 2, 3, …). For instance, for the sample in Figure 1, we obtain 1, 1, 1, 1, 1, 1, 1, 1, 1, 2, 1, 1, 1, 2, 3, 1, 2, 1, 1, …. While typical values of *ν* are one and two, a value larger than three can be seen sporadically. Subsequently, using a symbolization procedure [9,10,11,12], the sequence of numbers is expanded to 6-bit binary codes, *C*_1_ = 000000, *C*_2_ = 000001, … …, and *C*_64_ = 111111. It is interesting to note that replacing each compound in the text with an oscillator in a string, for the chain of the *ν*-*kanji* compounds, one can find an analogy with the Fermi-Pasta-Ulam-Tsingou (FPUT) model [13]. First, comparative analyses are conducted for 16 modern translations by 12 humans [14,15,16,17,18,19,20,21,22,23,24,25] and 4 machine translations using Google Translate and DeepL. It is interesting to investigate whether selecting a particular mode exhibits a behavior associated with ergodicity or not. In addition to the above modern-translated texts, 6 codices currently available for the classic are also analyzed, and their results are compared with those of modern translations. Unfortunately, the original has not been found yet. Eventually, a full comparison is performed among the 22 texts consisting of 16 modern translations (12 by humans and 4 by machines) and 6 codices.

## 2. Methods

**Figure 2:**
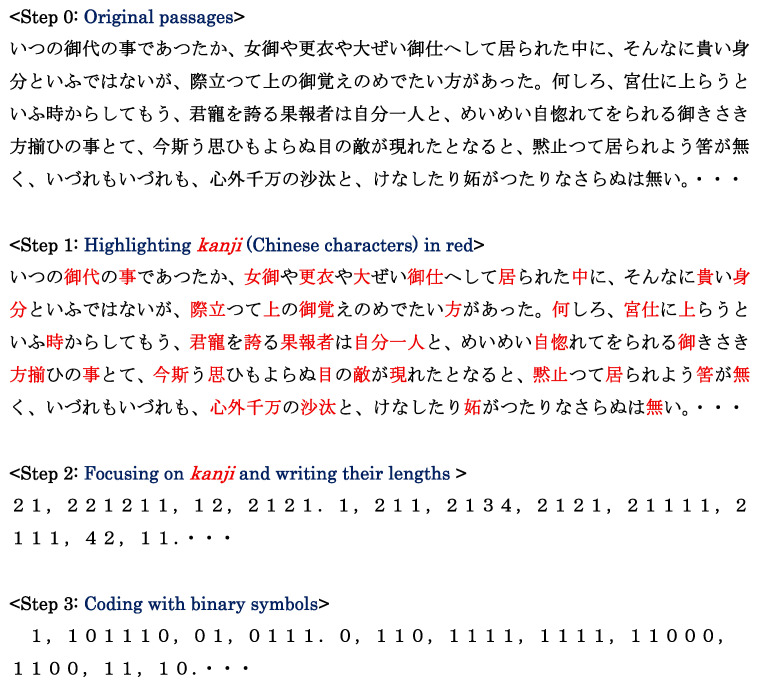
Procedure of producing binary sequence for passages sampled from a modern translation [14] of the opening texts in *Genji Monogatari* ‘*The Tale of Genji*’ originally written by Lady Murasaki (about 973―about 1014) in 1001 to 1005. The binary sequence in the final step is generated according to the rule of Equation (1).

**Figure 3:**
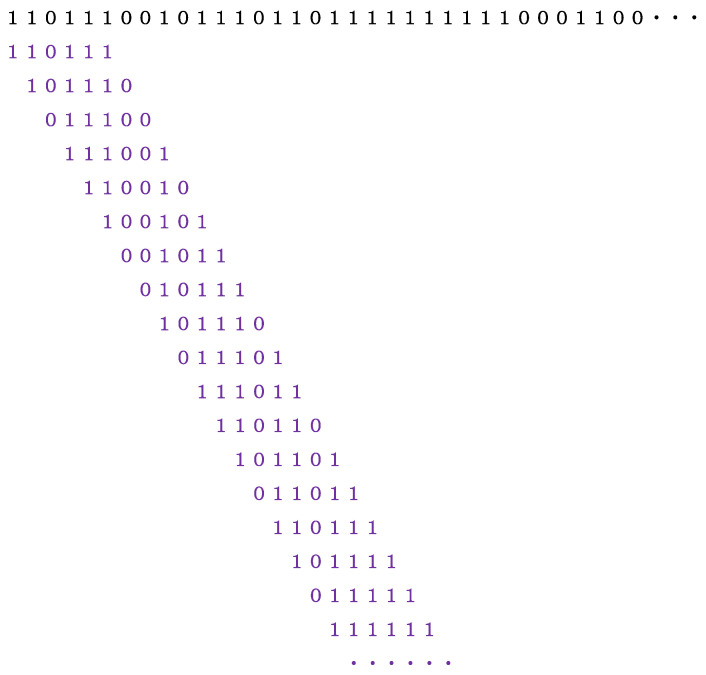
An illustration for explaining how to generate 6-bit binary codes from the opening data in Step 3 of Figure 2. Punctuation marks in Figure 2 are dropped.

The scheme of producing a binary sequence from a Japanese writing (Step 0) is given in Figure 2. Instead of the three kinds of characters being dealt with in the previous study [12], in this paper, we will confine ourselves solely to *kanji*. First, to generate the sequence of the length of *kanji* compounds, as in Figure 1, *kanji* are marked in red (Step 1). Subsequently, for each compound marked in red, the number of characters is counted (Step 2). Next, the binary sequence *s*_1_*s*_2_ … *s_n_*_−1_ is produced according to the rule (Step 3).

(1) $$s_{i}=\left\{\;\begin{array}{c} 0\;\;\;\;for\;x_{i+1}=x_{i}\\ 1\;\;\;for\;x_{i+1}\neq x_{i} \end{array}\right.$$

for *i* = 1, 2, …, *n* − 1, where *x_i_* (*i* = 1, 2, …, *n*) represents the number in Step 2, and *n* indicates the length of the entire binary sequence. Finally, one can obtain *n* − 5 binary codes from the sequence of Step 3. An example of how to obtain 6-bit binary sequences from the head part (34 bits from the start) in Step 3 is given in Figure 3.

From this rule, there arise 64 binary codes. Assuming a lexicographic order, we obtain *C*_1_ = 000000, *C*_2_ = 000001, … …, and *C*_64_ = 111111. To measure the diversity of the code spectra, Rényi entropy will be adopted

(2) $$H_{\alpha }\equiv H_{\alpha }\left(X\right)=\frac{1}{1-\alpha }log\left(\sum_{i=1}^{64}p_{i}^{\alpha }\right),$$

where *α* is a constant, and the 6-bit binary coding is implied. In the limit of *α* → 1, Equation (2) is reduced to the Shannon entropy

(3) $$H_{1}=\underset{\alpha \to 1}{\lim}H_{\alpha }=-\sum_{i=1}^{64}p_{i}\log p_{i},$$

while for *α* = 2 and *α* → ∞, respectively, Equation (2) is reduced to

(4) $$H_{2}=-log\sum_{i=1}^{64}p_{i}^{2},$$

(5) H∞≡limα→∞Hα=−log maxipi.

In the calculations that follow, we will use the normalized Rényi entropy.

(6) $$h_{\alpha }\equiv h_{\alpha }\left(X\right)=\frac{H_{\alpha }}{H_{0}},$$

with

(7) $$H_{0}=\log64=6\log2,$$

where $0\leq h_{\alpha }\leq 1$.

## 3. Results

**Figure 4:**
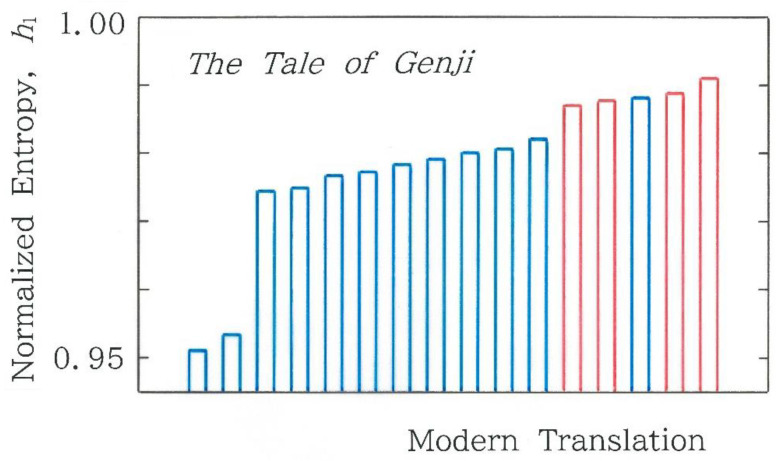
Comparison among normalized Shannon entropy for 16 modern translations of the first volume in *Genji Monogatari*, ‘*The Tale of Genji*’. The blue and red bars indicate the human and machine translations, respectively. The length of the sequence, *n*, is, in order from the left to right, 2904, 2128, 3265, 3810, 2983, 2283, 2590, 1377, 2475, 3326, 3173, 3373, 3195, 3620, 3434, and 3164.

In Figure 4, a comparison is made among the values of the normalized Shannon entropy for 16 modern translations of the first volume in *Genji Monogatari*, ‘*The Tale of Genji*’, where the blue and red bars indicate the human and machine translations, respectively. As for the machine translations (red bars) in Figure 4, we chose two translations from an English version by Waley [26] and from a French version by Sieffert [27]; in order from left to right in the red bars, one can see Google Translate from a French version (GF), DeepL from an English version (DE), DeepL from a French version (DF), and Google Translate from an English version (GE). As for the modern translations by humans, of the 12 translations [14,15,16,17,18,19,20,21,22,23,24,25], there is an exception (corresponding to Hayashi’s translation [25]) that is seen between DE and DF. The shortest bar on the left extreme corresponds to the translation by Tanizaki [15]. Evidently, there is a large discontinuity across the second [22] and third bars [23] from the left extreme (∆*h*_1_ = 0.021), in contrast to a smaller one across the fifth (GF) and sixth bars [18] from the right extreme (∆*h*_1_ = 0.0049). However, the latter is particularly worth noting in the context of stylometry and author attribution for human and machine translations.

**Figure 5:**
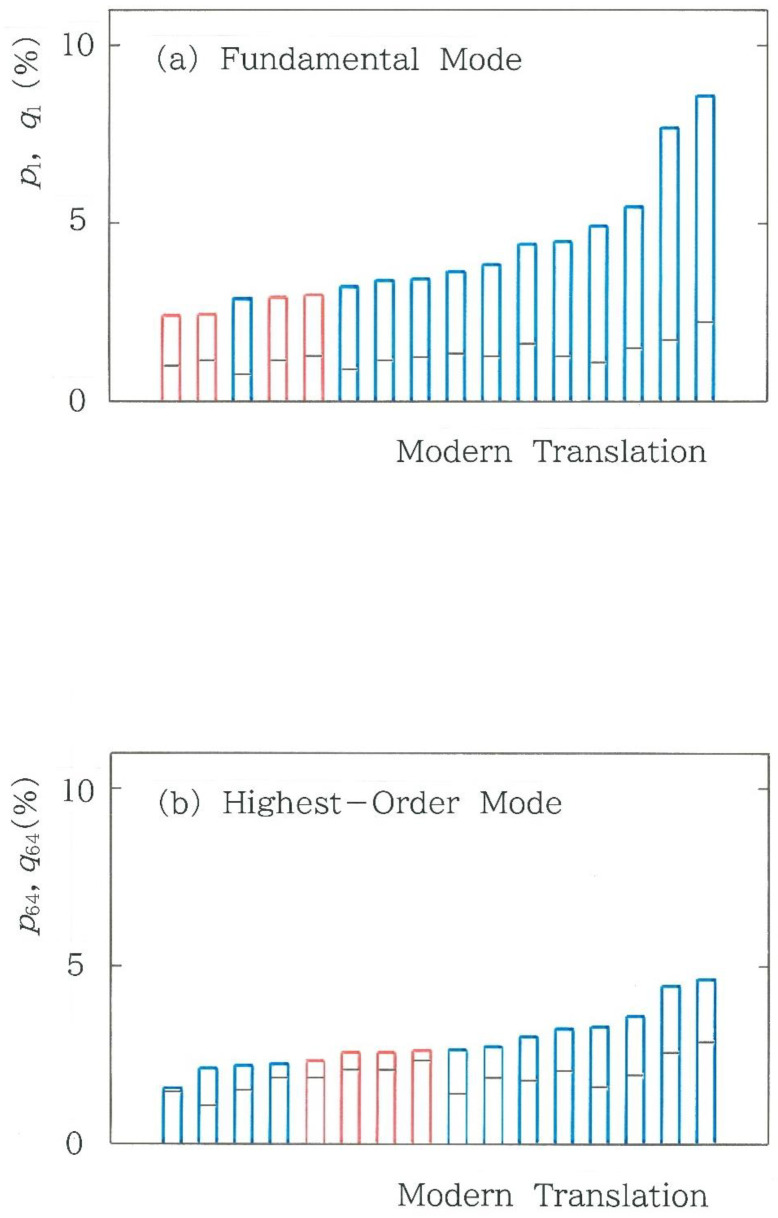
Comparison among the relative frequency of (**a**) the fundamental mode and (**b**) the highest-order mode for 16 modern translations of the first volume in *Genji Monogatari*. Here, *p_i_* denotes the relative value of the observed frequency, while *q_i_* denotes that of the expected frequency (*i* = 1, 64), which is highlighted using a black segment on each colored bar. The blue and red bars indicate the human and machine translations, respectively.

Subsequently, we will compare the relative frequencies of two dominant modes selected from the 64 modes. Here, in an analogy with a string of oscillators, we will replace the binary codes with a sort of modes. The results of the fundamental mode *C*_1_ = 000000 and the highest-order mode *C*_64_ = 111111 are shown in Figure 5a and Figure 5b, respectively, for the 16 modern translations of the first volume in *Genji Monogatari*. Here, *p_i_* denotes the relative value of the observed frequency, while *q_i_* denotes that of the expected frequency (*i* = 1, 64), the latter of which is highlighted using a black segment on each colored bar. The formulae of the latter are given as

(8) q1=1DM6, q64=1DN−M6, with D=N6.

Here, *M* and *N*, respectively, are the total of ‘0’ and the grand total of ‘0’ and ‘1’. Note that for the 6-bit coding, *N* = 6(*n* − 5). There are three features found in these plots: (1) Overall the observed frequencies of the fundamental mode (*p*_1_) are substantially higher than the expected ones (*q*_1_); (2) Except for a single case [14], the observed fundamental-mode frequencies of the human translations are higher than those of the machines; (3) In contrast to a steep curve in the fundamental mode, the ridgeline of the highest-order mode is relatively flat.

**Figure 6:**
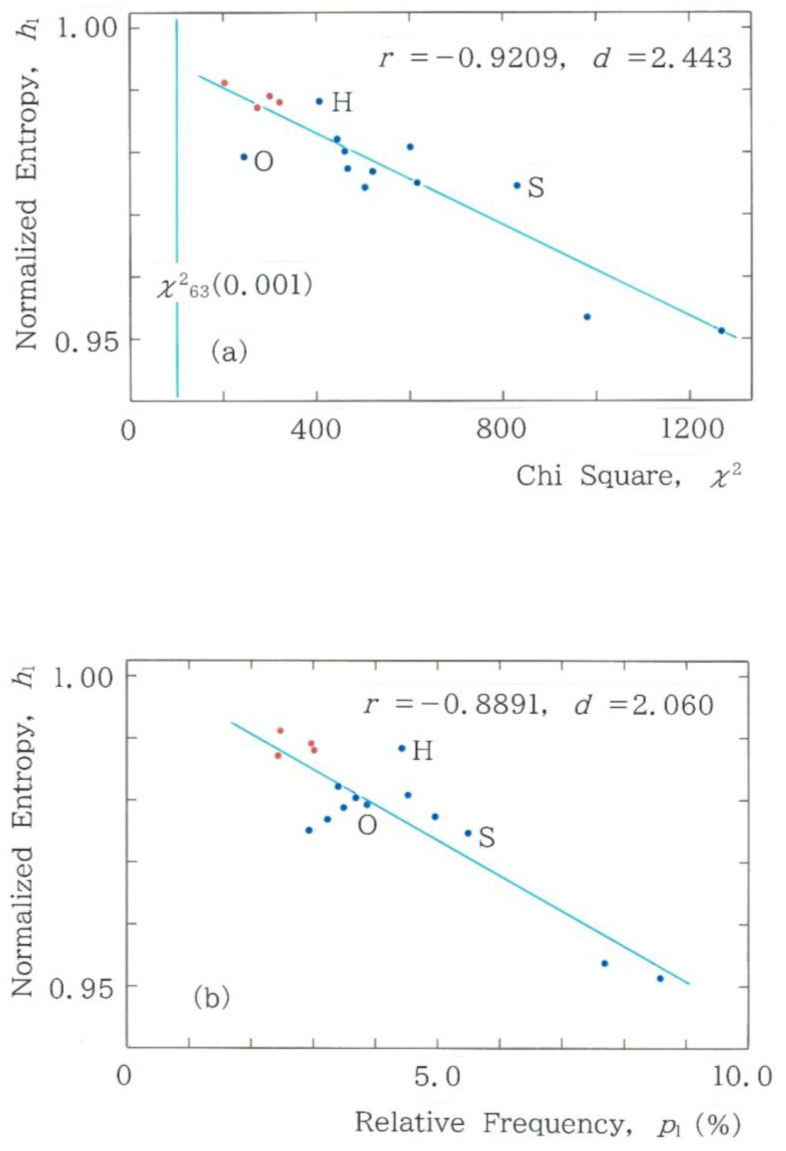
Dependence of the normalized Shannon entropy (Equation (6) with Equations (3) and (7)) on (**a**) the chi-square value (*y* = −3.652 × 10^−5^*x* + 0.9975) and on (**b**) the relative frequency of the first mode *C*_1_ = 000000 (*y* = −5.713 × 10^−3^*x* + 1.002). Results of the machine translations are highlighted in red. Initials H, O, and S denote modern translations by Hayashi [25], Ozaki [19], and Setouchi [23], respectively. Note that in (**a**), the vertical line in light blue stands for the critical chi-square value equal to 103.442 for 0.1% test being implied.

The results shown in Figure 4 and Figure 5a suggest that there are three clusters. To investigate this observation in more detail, in Figure 6a we show the dependence of the normalized Shannon entropy (Equation (6) for *α* = 1 with Equation (3)) on the chi-square value (See Appendix A), where dots of the 4 machine translations are highlighted in red and the vertical line in light blue stands for the critical chi-square value, $\chi_{63}^{2}$(0.001), which is equal to 103.442 for 0.1% test being implied. It can be seen that all translations exhibit $\chi^{2}>103.442$, the result of which allows one to conclude that the null hypothesis that claims stochasticity is rejected (*α* = 0.001). In consequence, it can be confirmed that our statistical model is far from ergodicity-like in the sense that a sort of ‘equal partition of energy’ does not occur. As for the clustering, due to the three outliers being highlighted with H, O, and S, one cannot confirm a distinct cluster in the entire cross-section. In an effort to reveal potential clusters, instead of the chi-square value, on the axis of abscissas, we will put the relative frequency of the fundamental mode *C*_1_ = 000000. The scattergram is shown in Figure 6b. A visual inspection of the cross-section can reveal three clusters, the sizes of which are found to be (4, 10, 2) from left to right. Note that the three outliers that were marked in Figure 6a belong to the intermediate cluster.

**Figure 7:**
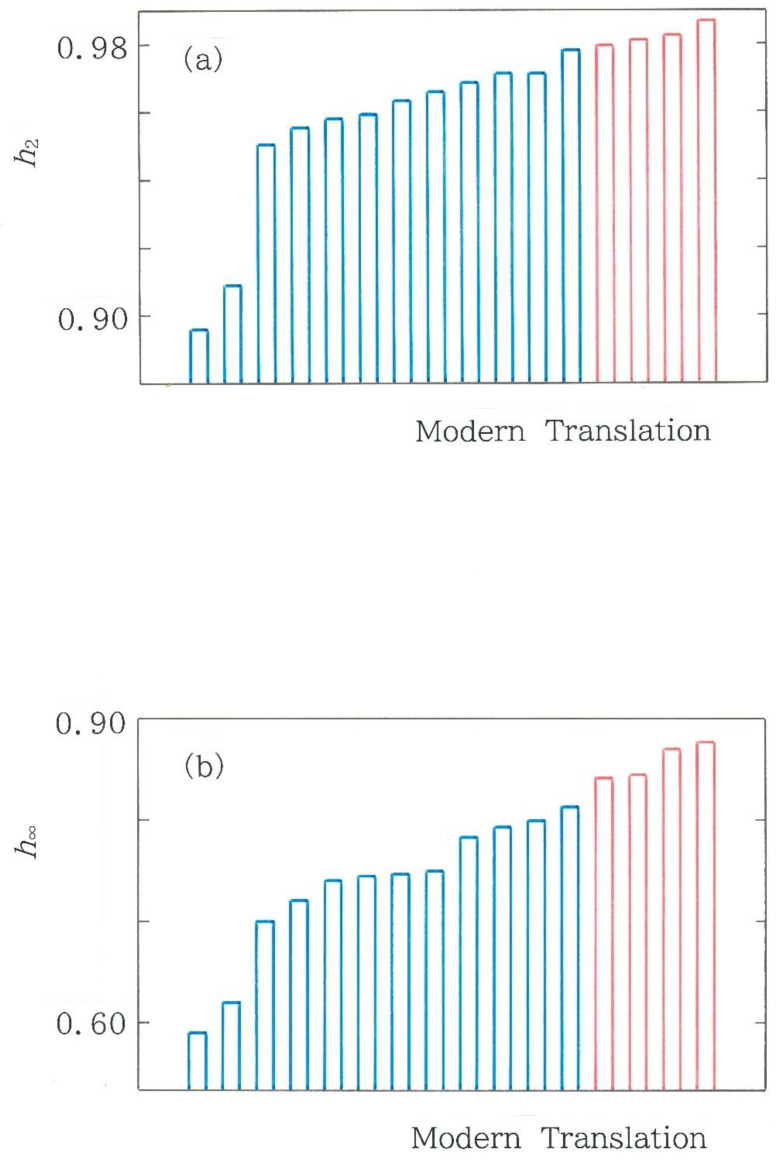
Comparison among normalized Rényi entropy *h_α_* for 16 modern translations of the first volume in *Genji Monogatari*. The blue and red bars indicate the human and machine translations, respectively. (**a**) *α* = 2. (**b**) *α* → ∞.

Finally, it seems interesting to make a comparison between the Shannon entropy corresponding to *α* → 1 and a couple of typical cases of Rényi entropy. The results for *α* = 2 and *α* → ∞ are shown in Figure 7a and Figure 7b, respectively, which should be compared with Figure 4. As in Figure 4, the blue and red bars indicate the human and machine translations, respectively. It can be observed that with increasing the value of *α*, the topography of the bars becomes smoother. In other words, the contrast between the human and machine translations tends to degenerate as the value of *α* increases. To this section, a postscript was added in Appendix B.

## 4. Discussion

### 4.1. Recurrence Phenomena of the Principal Mode

**Figure 8:**
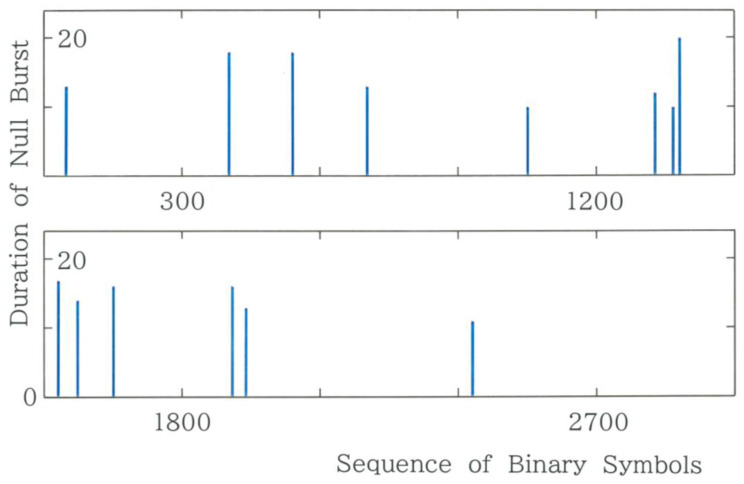
Sequential variation in the duration of null bursts for Tanizaki’s translation [15]. For instance, for the duration 20, there is a series of twenty zeroes. Note that null bursts with a duration less than 10 are omitted.

It is worth noting that our system shares a very complicated quasi-periodic behavior with the original FPUT system. To show a resemblance between the two, typical results of the sequential variation in the duration of the null burst are given in Figure 8. It can be observed that over the entire text the fundamental mode *C*_1_ = 000000 appears recurrently, suggesting at the same time the behavior far from equipartition of ‘energy’ to all possible modes.

### 4.2. Convergence of Entropy: Comparison of Partial and Full Texts

**Figure 9:**
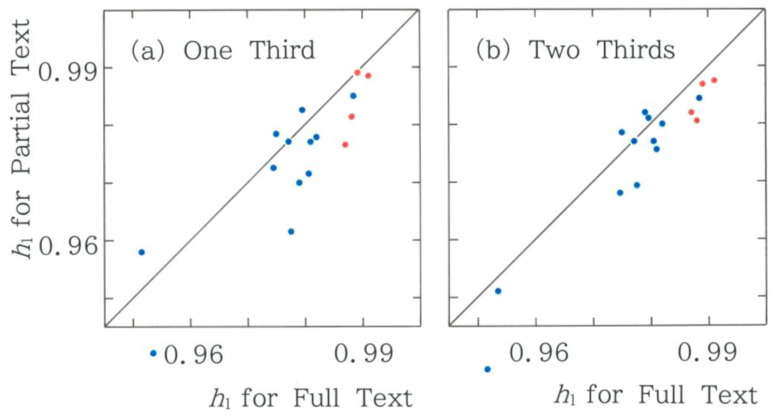
Relation between the normalized Shannon entropy of two partial texts and that of the full text. The blue and red dots indicate the human and machine translations, respectively. (**a**) One third of the full text (*r* = 0.8666, *d* = 2.223; *y* = 0.9488*x* + 0.04588). (**b**) Two-thirds of the full text (*r* = 0.9487, *d* = 2.001; *y* = 1.111*x* − 0.01120).

Although this paper deals with the entire text of the first volume in *Genji Monogatari*, it will be interesting to investigate the convergence behavior of entropies through comparison with the results for a few samples. The relation between the normalized Shannon entropy of two partial texts and that of the full text is displayed in Figure 9. In both samples, there are 12 points in the lower region (*y* < *x*), which amounts to 75%. The results reproduce a downward bias that could occur for shorter sequences.

### 4.3. Diversity Indices Other than Rényi Entropy

In addition to the Rényi entropy, there is another form of extension termed Tsallis entropy, *S_q_*, a normalization of which gives

(9) $$s_{q}\equiv s_{q}(X)=\frac{S_{q}}{\ln_{q}64},$$

with

(10) $$S_{q}\equiv \;S_{q}\left(X\right)=-\sum_{i=1}^{64}p_{i}\ln_{q}p_{i}.$$

**Figure 10:**
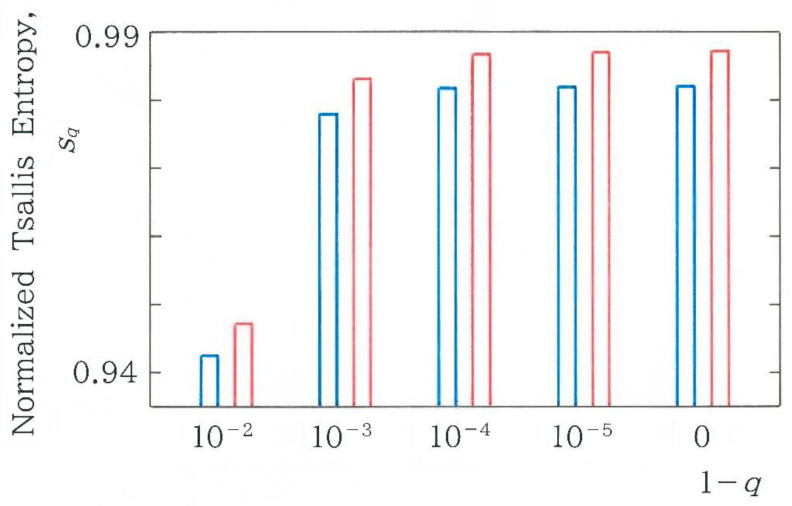
Dependence of normalized Tsallis entropy *s_q_* on 1 − *q*. The blue and red bars, respectively, indicate the modern translation by Enchi [18] and the translation by GF, i.e., Google Translate from a French version [27] of *Genji Monogatari*, ‘*Le Dit du Genji*’.

Here $0\leq s_{q}\leq 1$ and the *q*-logarithm in Equations (9) and (10) is defined with

(11) $$\ln_{q}y=-\frac{1-y^{1-q}}{1-q},$$

where it is implied that |1−q|≪1. Using the Box-Cox transform, one obtains $s_{q}\to h_{1}\;as\;q\to 1.$ We will apply the normalized Tsallis entropy to investigate whether the discontinuity across the human and machine translations can be expanded by varying the value of *q*. The dependence of the normalized Tsallis entropy *s_q_* on 1 − *q* is plotted in Figure 10. The blue and red bars, respectively, indicate the modern translation by Enchi [18] and the translation by GF, i.e., Google Translate from a French version [27] of *Genji Monogatari*, ‘*Le Dit du Genji*’. In Figure 4, these coincide with the sixth and fifth bars, respectively, from the right extreme. Calculation of the difference (i.e., discontinuity) in the normalized entropy yields ∆*s_q_* = 0.00468, 0.00491, 0.00493, 0.00494, and 0.00494 for 1 − *q* = 10^−2^, 10^−3^, 10^−4^, 10^−5^, and 0, respectively. It is found that as the value of *q* increases, the difference increases slightly. In conclusion, using the Shannon entropy with *q* = 1, one can detect most distinctly the difference between the human and machine translations.

In Figure 4 and Figure 7, for the three kinds of normalized entropies, a comparison was made among 16 modern translations, but there is a diversity metric without using the concept of entropy. To investigate the potential of the metric, in addition to the entropy, we consider Simpson’s diversity index 1 − *λ* with *λ* given by [28]

(12) λ=∑i=164fi2n−52=∑i=164fi (fi−1)(n−5)(n−6). 

**Figure 11:**
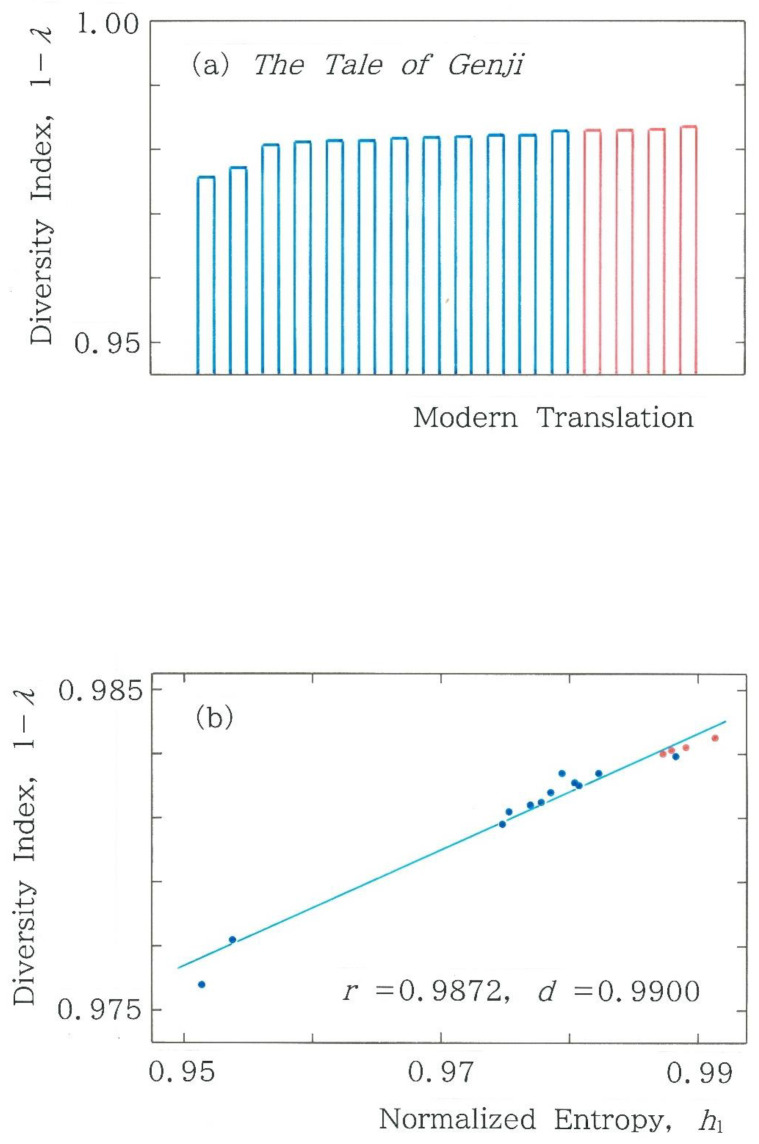
(**a**) Comparison among Simpson’s diversity indices for 16 modern translations of the first volume in *Genji Monogatari*, ‘*The Tale of Genji*’. The blue and red bars indicate the human and machine translations, respectively. (**b**) Dependence of Simpson’s diversity index on the normalized Shannon entropy (*y* = 0.1827*x* + 0.8028). The blue and red dots indicate the human and machine translations, respectively.

Here, 0 ≤ 1 − *λ* ≤ 1 and *f_i_* = (*n* − 5) *p_i_* (*i* = 1 to 64) for the 6-bit binary coding being implied. In Figure 11a comparison is made among Simpson’s diversity indices for 16 modern translations of the first volume in *Genji Monogatari*. As in Figure 4 and Figure 7, the blue and red bars indicate the human and machine translations, respectively. Evidently, the envelope of the bars becomes substantially flatter than those seen for the Rényi entropies (Figure 4 and Figure 7), and at the same time, the discontinuity across the human and machine translations vanishes (∆(1 − *λ*) ≒ 0). Subsequently, the relation between Simpson’s diversity index and the normalized Shannon entropy is shown in Figure 11b, where there are three clusters, (2, 9, 5), from left to right; an isolated blue dot in the third cluster corresponds to the translation by Hayashi [25]. It should be noted here that although the modulus of the correlation coefficient, |*r*|, exceeds those seen in Figure 6a,b, the value of the Durbin-Watson ratio, *d*, becomes smaller than the lower critical value *d_L_* = 1.10 (*α* = 0.05) in the test of serial residual correlation.

### 4.4. Scattergrams for Hellinger Distances from Machine Translations

**Figure 12:**
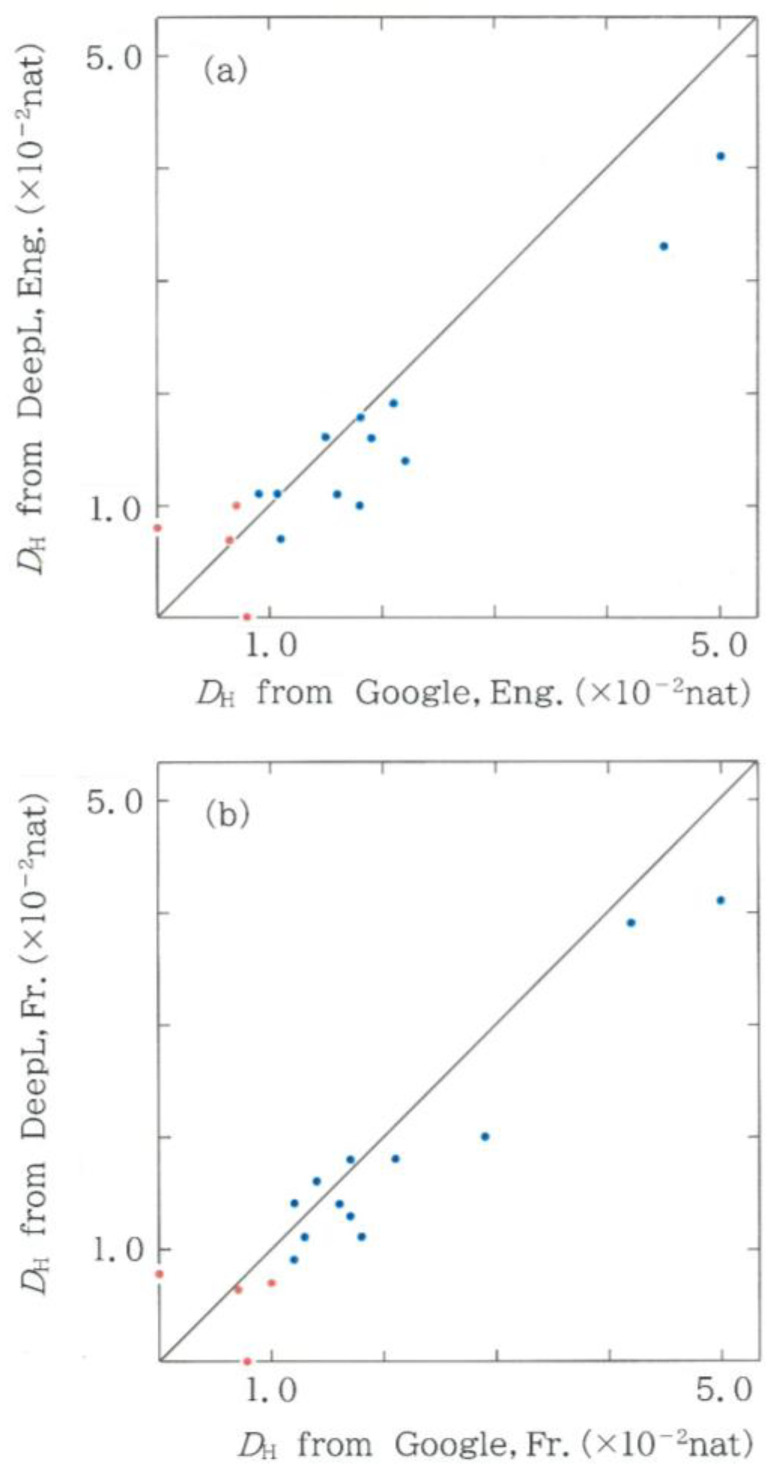

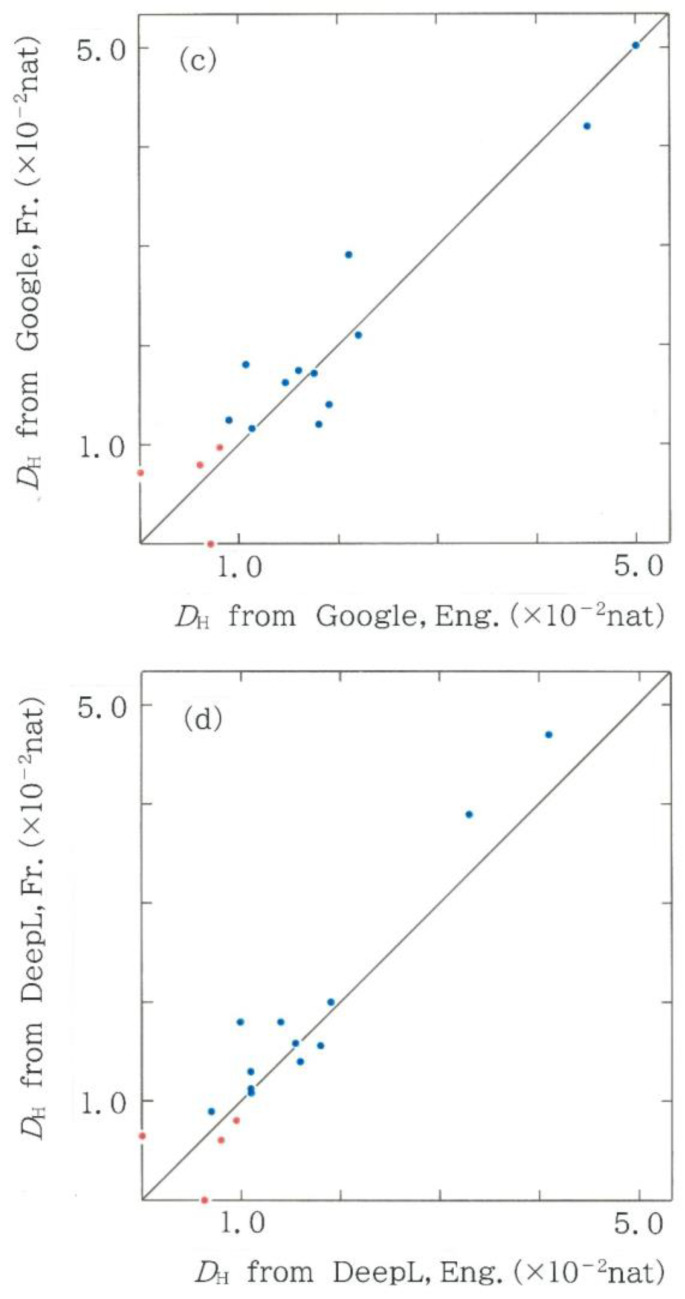
The relation between two Hellinger distances *D_H_*_’_’s for 16 modern translations of the first volume in *Genji Monogatari*, ‘*The Tale of Genji*’. The blue and red dots indicate the human and machine translations, respectively. (**a**) Hellinger distance from translation by DeepL for an English version (DE) versus that by Google Translate for the same version (GE). (**b**) Hellinger distance from translation by DeepL for a French (DF) version versus that by Google Translate for the same version (GF). (**c**) Hellinger distance from translation by Google Translate for a French version (GF) versus that by the same machine-translation device for an English version (GE). (**d**) Hellinger distance from translation by DeepL for a French version (DF) versus that by the same device for an English version (DE).

As for machine translation devices, we have employed Google Translate and DeepL. It appears interesting to compare their translation abilities into modern Japanese. To this end, of the six combinations (choosing two from four) we will focus on four significant cases: (1) DeepL from English (DE) versus Google Translate from the same language (GE), (2) DeepL from French (DF) versus Google Translate from the same language (GF), (3) Google Translate from French (GF) versus the same device from English (GE), and (4) DeepL from French (DF) versus the same device from English (DE). In Figure 12a–d, respectively, for the 16 modern translations of the first volume in *Genji Monogatari*, dots are plotted between two Hellinger distances, the definition of which is given by

(13) $$D_{H\;}=\sum_{i=1}^{64}(\sqrt{p_{i}}-\sqrt{q_{i}\;}{)}^{2},$$

where *p_i_* and *q_i_* (*i* = 1, 2, …, 64) are the relative frequencies in question, and the unit of the distance is nat, which abbreviates natural unit. The blue and red dots represent the human and machine translations, respectively. In each scattergram, counting the number of blue dots in the upper (*y* > *x*) and lower (*y* < *x*) sections enables one to evaluate the relative performance of the two devices. Denoting the contents of dots in the two regions as (@, @), we obtain (3, 9) for Figure 12a (DE versus GE), which suggests that for English, the performance of DeepL is higher than that of Google Translate. Similar counting for Figure 12b (DF versus GF) shows a result exactly identical to Figure 12a. In summary, it has been confirmed that for both English and French, DeepL possesses translation abilities higher than Google Translate. Next, instead of a comparison between two machine-translation devices, for each device, we will investigate which language works better. First, for Google Translate, from Figure 12c (GF versus GE), we obtain (7, 5), indicating that for Google Translate, the performance for English is slightly higher than for French. For DeepL plotted in Figure 12d (DF versus DE), the bias between the two languages is expanded up to (9, 3). In consequence, it seems that, in general, machine translations from English texts work better than those from French.

### 4.5. Dimensionality Reductions: A Stability Analysis

**Figure 13:**
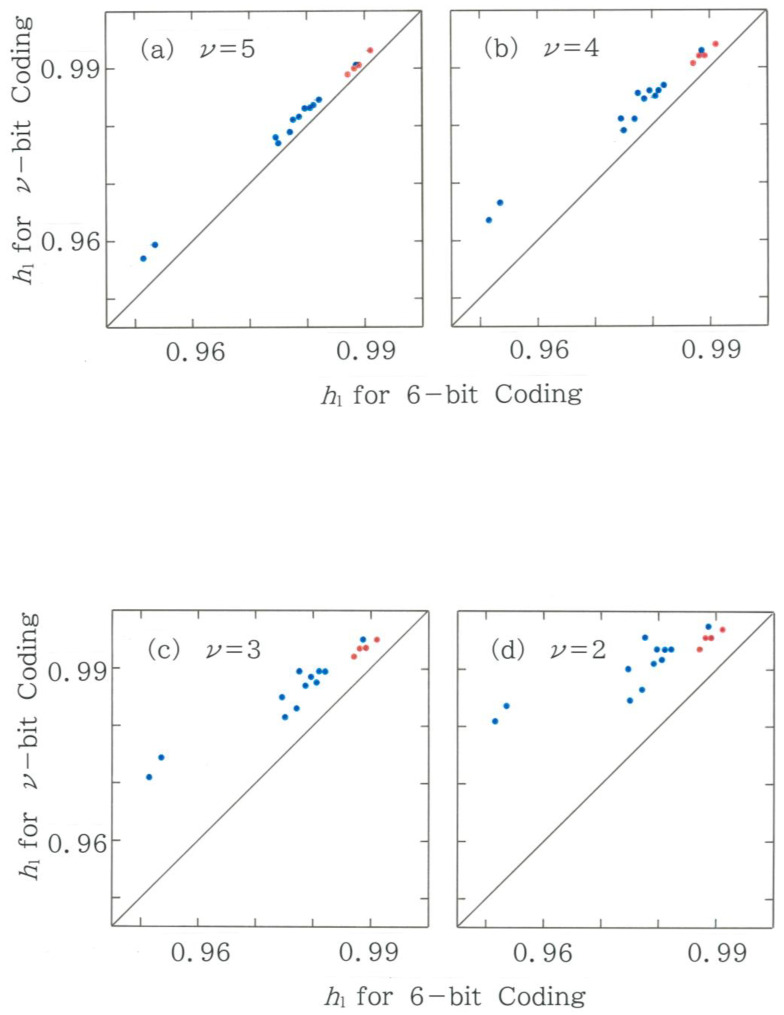
Relation between the normalized Shannon entropy of reduced codes and that of the original code. The blue and red dots indicate the human and machine translations, respectively. (**a**) 5-bit coding (*r* = 0.9986, *d* = 1.885; *y* = 0.8963*x* + 0.1043). (**b**) 4-bit coding (*r* = 0.9915, *d* = 1.843; *y* = 0.7563*x* + 0.2442). (**c**) 3-bit coding (*r* = 0.9689, *d* = 1.868; *y* = 0.5934*x* + 0.4067). (**d**) 2-bit coding (*r* = 0.8775, *d* = 1.908; *y* = 0.3862*x* + 0.6136).

While in this paper we employ 6-bit coding, it will be interesting to make a comparison with other coding methods. Computed results of the normalized Shannon entropy are shown in Figure 13, where 4 kinds of coding bits less than 6 are chosen and compared with the original 6-bit coding. Note that coding bits higher than six are not applicable to the length of our text. From the four scattergrams, it is found that, overall, the entropy shows stability in how to symbolize the sequence, as a relatively high correlation (*r* = 0.9) is preserved even with 2-bit coding. It seems that the upward shift of the dots, which results in their sharp concentration in the upper half region (*y* > *x*), arises from an averaging effect due to the coarse graining.

**Figure 14:**
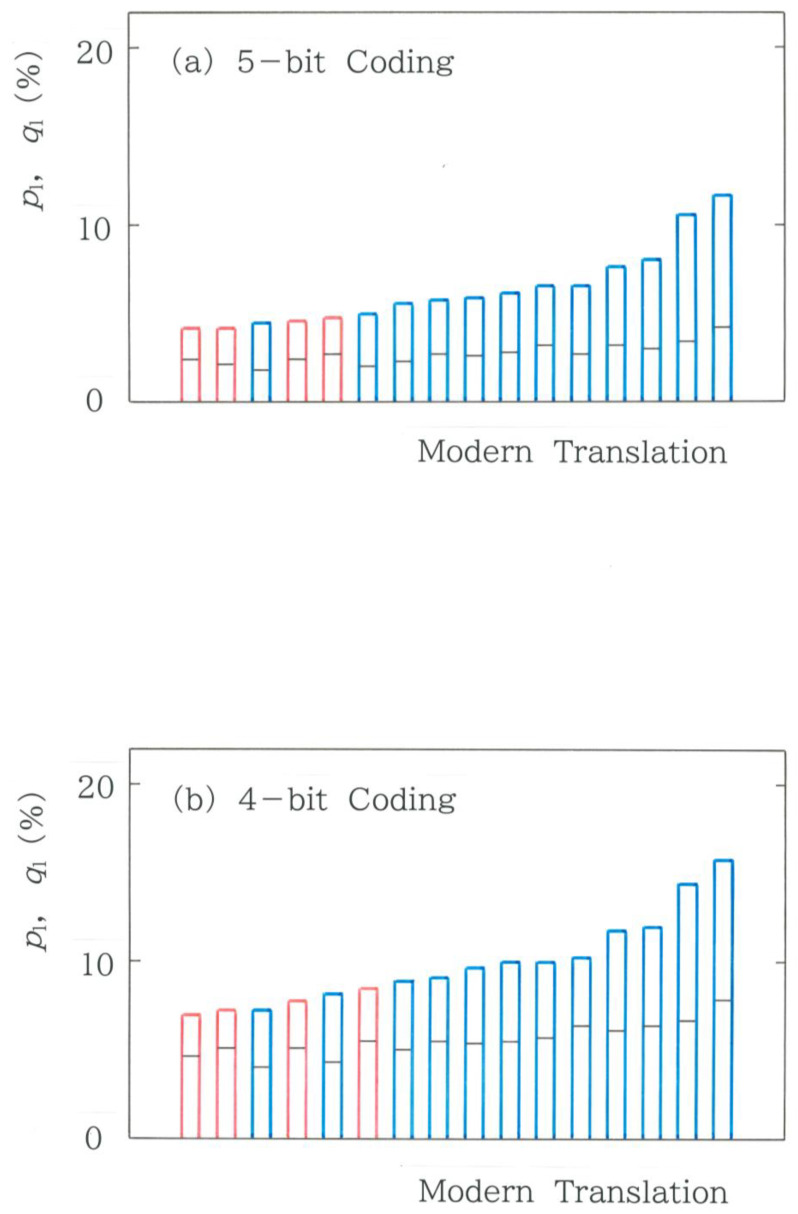

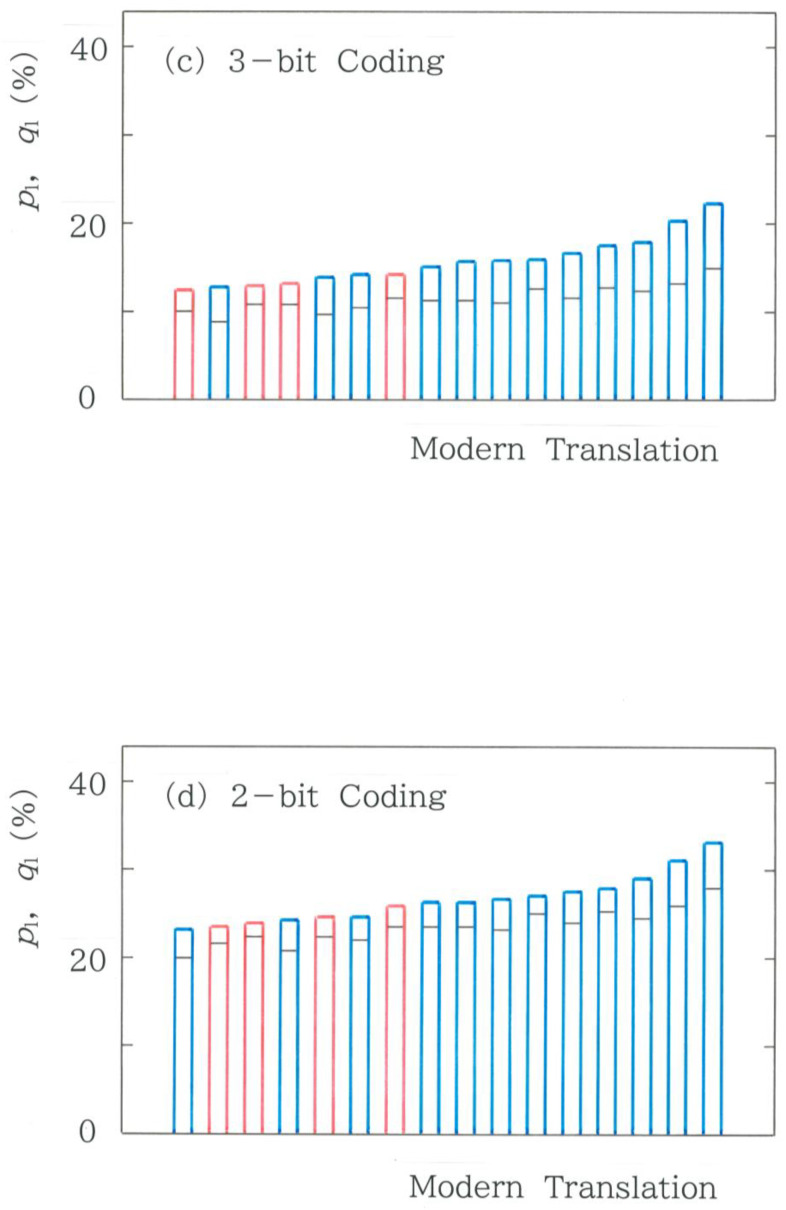
Comparison among relative frequencies of the fundamental mode for 16 modern translations of the first volume in *Genji Monogatari*. As in Figure 5a, *p*_1_ denotes the relative value of the observed frequency, while *q*_1_ denotes that of the expected frequency, which is highlighted using a black segment on each colored bar. The blue and red bars indicate the human and machine translations, respectively. (**a**) 5-bit coding. (**b**) 4-bit coding. (**c**) 3-bit coding. (**d**) 2-bit coding.

Subsequently, we consider the effect of the dimensionality reduction on the relative frequencies of the fundamental mode. Computed results are displayed in Figure 14. Here, as in Figure 5a, for each symbolization, a comparison is made between the surveyed and expected frequencies for 16 modern translations of the first volume in *Genji Monogatari*. It is obvious in the plots that, irrespective of the method of coding, remarkable concentration is seen on the fundamental mode. That is, for all cases, the inequality *p*_1_ > *q*_1_ is preserved with no exception.

### 4.6. Comparison Among Codices

**Figure 15:**
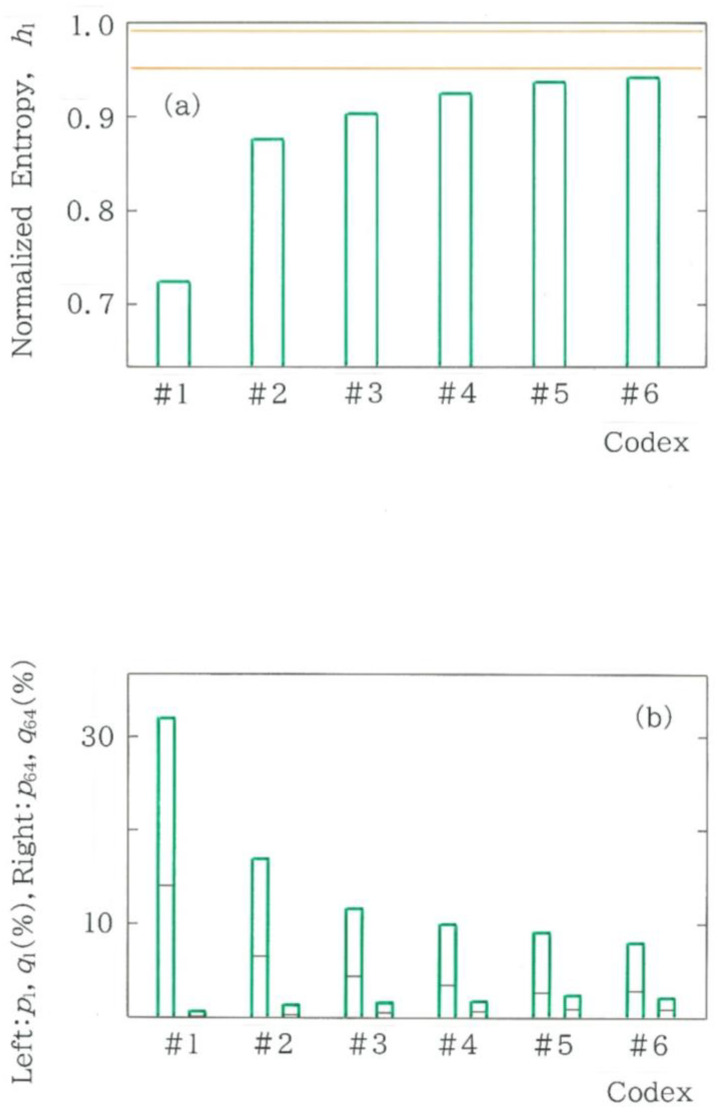
Comparison among (**a**) normalized Shannon entropy and (**b**) relative frequency for 6 codices of the first volume in *Genji Monogatari*, ‘*The Tale of Genji*’. The length of the sequence, *n*, is, in order from Codex #1 to #6, 1545, 1749, 1674, 2357, 1520, and 1408. In (**a**), the parallels in orange stand for the range of the modern translations, 0.9513 ≤ *h*_1_ ≤ 0.9912, which was obtained from Figure 4. In (**b**), *p_i_* denotes the relative value of the observed frequency, while *q_i_* denotes that of the expected frequency (*i* = 1, 64), which is highlighted using a black segment on each green bar.

So far, we have concentrated on the modern translations of the first volume in *Genji Monogatari*. It appears very interesting to conduct a similar analysis for the original and to make a comparison with the results of the modern translations. Although, unfortunately, the original text is not currently available, there are several codices available instead. In Figure 15a, we show the results of the normalized Shannon entropy for six codices #1 to #6 [14,17,20,29,30,31] of the first volume of the classic. The length of the sequence, *n*, is, in order from Codex #1 to #6, 1545, 1749, 1674, 2357, 1520, and 1408. The parallel lines in orange highlight the range of the modern translations, 0.9513 ≤ *h*_1_ ≤ 0.9912, in Figure 4. It is interesting to see that entropies of the codices do not exceed the lower bound of the modern translations. In Figure 15b, *p_i_* denotes the relative value of the observed frequency, while *q_i_* denotes that of the expected frequency (*i* = 1, 64), which is highlighted using a black segment on each green bar. The results of Figure 15b are consistent with those of Figure 15a. In particular, the exceptionally low entropy observed for Codex #1 [17] can be explained by the intense concentration on the fundamental mode *C*_1_, in contrast to the negligible frequency on the highest-order mode *C*_64_.

### 4.7. Full Comparison Among Modern Translations and Codices

**Figure 16:**
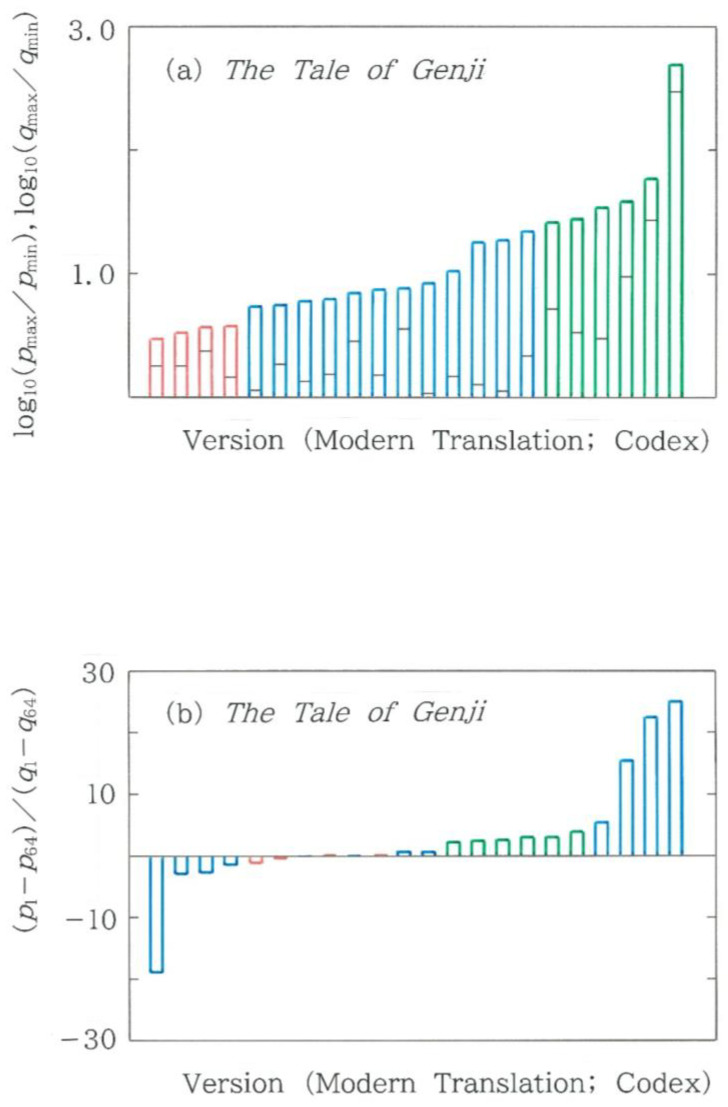
Comparison among metrics for 16 modern translations and 6 codices. Red and blue bars, respectively, indicate modern translations by humans and machine-translation devices (Google Translate and DeepL), while green bars indicate texts in codices. (**a**) Common logarithm of the highest frequency divided by the lowest one. Note that the black segments on each bar indicate the expected values. (**b**) Ratio *ρ* of the difference in the relative frequency, *p*_1_ − *p*_64_, versus the one for the expected value, *q*_1_ − *q*_64_. Of the 22 bars, the sixth to tenth bars from the left extreme appear too short to discriminate, which are reproducible with red, blue, red, blue, and red, respectively.

In Figure 4, Figure 5, Figure 6, Figure 7, Figure 8, Figure 9, Figure 10, Figure 11, Figure 12, Figure 13 and Figure 14, we compared 16 modern translations, while in Figure 15, a comparison has been made among 6 codices. Below, we will make a full comparison among the modern translations and codices. First, to detect a sort of ‘blowup’ that was predicted for a nonlinear localization [32], we consider the highest frequency divided by the lowest one. The results are shown in Figure 16a. Note that the ordinate is scaled with the common logarithm, and the black segments on each bar indicate the expected values. Evidently, the entire bar can be divided very clearly into three groups: modern translations by machines (in red), those by humans (in blue), and codices (in green). It seems that of the 22 cases, only the codex on the right extreme exhibits a feature close to the blowup. Lastly, to reveal a human bias in the writings, we consider the ratio *ρ* of the difference in the relative frequency, *p*_1_ − *p*_64_, versus the one for the expected value, *q*_1_ − *q*_64_. That is,

(14) $$\rho =\frac{p_{1}-p_{64}}{q_{1}-q_{64}}.$$

The results are displayed in Figure 16b. Here it should be stressed that (1) overall the moduli |*ρ*| of the modern translations by humans (in blue) are larger than those of the machine translations (in red) as well as codices (in green), (2) the moduli of the machine translations (corresponding to, from the left extreme, the fifth, sixth, eighth, and tenth bars in red) are extremely small (0.1 < |*ρ*| < 1.2), and (3) the six codices (the fifth to tenth bars from the right extreme) preserve the values of *ρ* (2.3 < *ρ* < 4.0) that are intermediate between the other groups. Lastly, in Figure 16b, it is worth emphasizing that, in several cases, the ratio values become negative (*ρ* < 0). Because *p*_1_ > *p*_64_ is confirmed in all cases, the ratios can become negative when *q*_1_ < *q*_64_. Such an anomaly might remind one of the ‘population inversion’ in lasers.

## 5. Conclusions

For modern translations of a famous novel written in an archaic style of Japanese, we have conducted an analysis of a chain of *kanji* compounds in the text. Specifically, for the first volume of *Genji Monogatari*, ‘*The Tale of Genji*’, using Rényi entropy, we have investigated a modal competition in the sequence of their numbers, where each number (*ν*) coincides with that in a *kanji* compound. Subsequently, the sequence of numbers has been transformed to 6-bit binary codes, *C*_1_ = 000000, *C*_2_ = 000001, … …, and *C*_64_ = 111111, each of which can be regarded as a sort of vibrating mode. That is, replacing each compound in the text with an oscillator in a string, for the chain of the *ν*-*kanji* compounds, one can see a similarity of form between the sequence of the numbers and the sequence of the amplitudes of oscillators in the Fermi-Pasta-Ulam-Tsingou (FPUT) problem. First, on the basis of a chi-square test (*α* = 0.001), analyses of the entire modern translation by 12 humans and 4 machines have shown a property far from stochastic. Next, a comparison between the human and machine translations has shown that overall, the entropy of the latter is higher than that of the former because machines are subjected to neither a psychological bias nor an inspection by editors. Computed results of the 6 codices of the classic have shown that the values of entropy become lower than those obtained for the modern translations, and at the same time, the non-stochastic behavior is preserved. Irrespective of the kinds of texts, the fundamental mode *C*_1_ = 000000 in our sequence has been found to be extremely stable and to possess a property bearing a remote resemblance to a soliton [33] predicted in the original FPUT model. While this study has revealed the pronounced dominance of the fundamental mode, exploring possibilities of dominance for other kinds of modes in a great deal of Japanese writings will be of theoretical interest.

So far, in an effort to make available an automatic metric for machine-translation quality, powerful devices such as BLEU [34] and COMET [35,36] have been developed along with a novel quantitative framework combining rank-frequency statistics with time-reversal asymmetry to assess translation quality [37]. The findings in this paper suggest that the present method can also provide an additional tool useful for stylometry and author attribution. In other words, it could detect whether a certain text in question was written by a human or generated with the aid of artificial intelligence. Indeed, through comparison between several diversity indices, the capabilities of Shannon’s entropic measure have been confirmed. Lastly, it should be mentioned that there are limitations to our method. First, in contrast to the original FPUT system, the dynamics in the humanities depends on uncertain factors, which do not allow one to produce a genuine dynamical equation. That is, the analogy inspired by the system is phenomenal and could not be taken as a direct physical correspondence. Second, while the coding rule of Equation (1) focuses on a variation of the length in the sequence of *kanji* compounds, it could not deal with the magnitude of the variation. There is further work ahead.

## Data Availability

The raw data supporting the conclusions of this article will be made available by the author without undue reservation.

## Appendix A. Calculation of Chi-Square Value

In order to implement a nonparametric test, the chi-square value is useful:

$$\chi^{2}=\;\sum_{i=1}^{64}\frac{(f_{i}-F_{i}{)}^{2}}{F_{i}}.$$

Here, *f*_i_ and *F*_i_ (*i* = 1 to 64) represent the surveyed and expected frequencies, respectively. Note that to avoid singularity, *F_i_* ≠ 0 unless *f_i_* vanishes. The relations with the relative frequencies in the main text are *p_i_* = *f_i_*/(*n* − 5) and *q_i_* = *F_i_*/(*n* − 5). With a theory of combinatorial probability, the expected frequencies can be calculated with the formulae

F1=n−5DM6,

Fi=n−56DM5N−M1 for i=2, 3, 5, 9, 17, 33,

Fi=n−515DM4N−M2 for i=4, 6, 7, 10, 11, 13, 18, 19, 21, 25, 34, 35, 37, 41, 49,

Fi=n−520DM3N−M3 for i=8, 12, 14, 15, 20, 22, 23, 26, 27, 29, 36, 38, 39, 42, 43, 45, 50, 51, 53, 57,

Fi=n−515DM2N−M4 for i=16, 24, 28, 30, 31, 40, 44, 46, 47, 52, 54, 55, 58, 59, 61,

Fi=n−56DM1N−M5 for i=32, 48, 56, 60, 62, 63,

F64=n−5DN−M6,

where the formula of *D* is given by Equation (8) in the main text.

## Appendix B. Addendum to Section 3

After the submission of this article, the author found an additional modern translation of *Genji Monogatari*. Specifically, in 2023, Kakuta published an updated version [38], which has shown that *h*_1_ = 0.9817, $\chi^{2}=504.577$, and *p*_1_ = 5.08%. The results indicate that Kakuta’s dots belong to the second cluster in Figure 6. Other entropies are *h*_2_ = 0.9614 and *h*_∞_ = 0.7166. The dots in Figure 12 are *D*_H_ = 1.18 × 10^−2^ (nat) for DE, *D*_H_ = 1.29 × 10^−2^ (nat) for DF, *D*_H_ = 1.52 × 10^−2^ (nat) for GE, and *D*_H_ = 1.67 × 10^−2^ (nat) for GF. Consequently, Kakuta’s dots will be seen in the lower regions of Figure 12a,b, and in the upper regions of Figure 12c,d.
